# Cationic Serine-Based Gemini Surfactant:Monoolein Aggregates as Viable and Efficacious Agents for DNA Complexation and Compaction: A Cytotoxicity and Physicochemical Assessment

**DOI:** 10.3390/jfb15080224

**Published:** 2024-08-13

**Authors:** Isabel S. Oliveira, Sandra G. Silva, Andreia C. Gomes, M. Elisabete C. D. Real Oliveira, M. Luísa C. do Vale, Eduardo F. Marques

**Affiliations:** 1 CIQUP (Centro de Investigação em Química da Universidade do Porto), IMS (Institute of Molecular Sciences), Departamento de Química e Bioquímica, Faculdade de Ciências, Universidade do Porto, Rua do Campo Alegre s/n, 4169-007 Porto, Portugal; 2 LAQV-REQUIMTE (Laboratório Associado para a Química Verde-Rede Química e Tecnologia), Departamento de Química e Bioquímica, Faculdade de Ciências, Universidade do Porto, Rua do Campo Alegre s/n, 4169-007 Porto, Portugal; sandra.silva@fc.up.pt (S.G.S.); mcvale@fc.up.pt (M.L.C.d.V.); 3 CBMA (Centro de Biologia Molecular e Ambiental), Departamento de Biologia, Campus de Gualtar, Universidade do Minho, 4710-057 Braga, Portugal; agomes@bio.uminho.pt; 4 CFUM (Center of Physics), Departamento de Física, Universidade do Minho, Campos de Gualtar, 4710-057 Braga, Portugal; beta@fisica.uminho.pt

**Keywords:** gemini surfactants, serine, lipoplex, DNA complexation/compaction, non-viral gene delivery, chain length, helper lipid, monoolein

## Abstract

Cationic gemini surfactants have emerged as potential gene delivery agents as they can co-assemble with DNA due to a strong electrostatic association. Commonly, DNA complexation is enhanced by the inclusion of a helper lipid (HL), which also plays a key role in transfection efficiency. The formation of lipoplexes, used as non-viral vectors for transfection, through electrostatic and hydrophobic interactions is affected by various physicochemical parameters, such as cationic surfactant:HL molar ratio, (+/−) charge ratio, and the morphological structure of the lipoplexes. Herein, we investigated the DNA complexation ability of mixtures of serine-based gemini surfactants, (nSer)_2_N5, and monoolein (MO) as a helper lipid. The micelle-forming serine surfactants contain long lipophilic chains (12 to 18 C atoms) and a five CH_2_ spacer, both linked to the nitrogen atoms of the serine residues by amine linkages. The (nSer)_2_N5:MO aggregates are non-cytotoxic up to 35–90 µM, depending on surfactant and surfactant/MO mixing ratio, and in general, higher MO content and longer surfactant chain length tend to promote higher cell viability. All systems efficaciously complex DNA, but the (18Ser)_2_N5:MO one clearly stands as the best-performing one. Incorporating MO into the serine surfactant system affects the morphology and size distribution of the formed mixed aggregates. In the low concentration regime, gemini–MO systems aggregate in the form of vesicles, while at high concentrations the formation of a lamellar liquid crystalline phase is observed. This suggests that lipoplexes might share a similar bilayer-based structure.

## 1. Introduction

The use of oligonucleotides (DNA, antisense oligonucleotides, mRNA, and siRNA) as therapeutic drugs to replace missing genes, to correct defective genes, or to downregulate aberrant gene expression stands as a disruptive approach to treating new diseases [1,2]. A successful gene therapy requires, however, the development of efficient nucleic acid (NA) delivery systems that act as a protective capsule for the NA cargo and prevent enzymatic degradation until the delivery within target cells [3,4,5]. In recent decades, several nanomaterial-based platforms, showing an enhanced therapeutic efficacy and reduced side effects, have been developed as non-viral gene delivery vectors [6]. These vehicles include micelles, liposomes, nano/microcapsules, and nano/microparticles made from biocompatible hydrophobic polymers or copolymers of natural or synthetic origin [5,7,8,9,10]. Each carrier type presents advantages and drawbacks, and its efficiency depends on specific characteristics presented by the different cell types, including the lipid composition of the cytoplasmic membrane [11]. Thus, choosing the right delivery system requires considering several relevant factors, such as acute and chronic unspecific toxicity, biodegradability of the nanomaterials, and the elimination processes of intact or metabolized chemicals.

**Figure 1:**
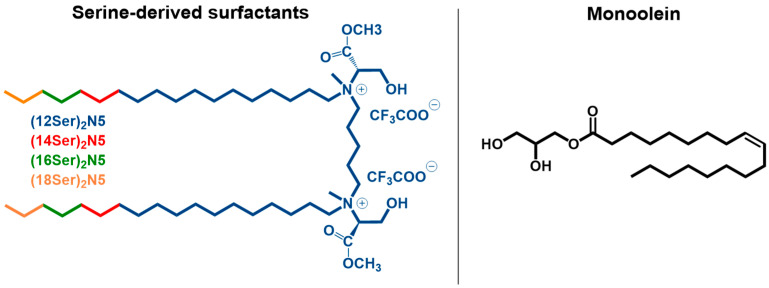
Molecular structure of the serine-based bis-quat surfactants used in this work and monoolein (MO).

Double-tailed cationic gemini surfactants, presenting two polar headgroups connected by a covalent spacer, have been successfully used as building blocks for NA delivery systems [12,13,14,15]. Most of the reports have focused on compounds with quaternary ammonium headgroups and linear aliphatic tails, the so-called bis-quats, and more recently on bio-inspired amphiphiles specially designed to overcome the toxicity issues presented by the former compounds [16,17,18,19,20]. The complexation of NA by cationic amphiphiles results mainly from electrostatic interactions between the phosphate backbone of the nucleotides and the positively charged headgroups of the surfactants. Nevertheless, cationic surfactants, when used *per se*, possess a high positive charge density, which imparts to them an unfavorable cytotoxicity, thus hampering their widespread use for NA delivery in vivo. To offset this excess of charge, neutral or zwitterionic lipids are almost invariably added to the formulation. Besides lowering charge density, the inclusion of helper lipids confers to the system higher stability and enhances membrane perturbation and fusion, thus increasing the transfection efficiency [17,21]. Lipids, such as 1,2-dioleoyl-*sn*-glycero-3-phosphoethanolamine (DOPE), phosphatidycholines, and cholesterol are often used as helper lipids in plasmid DNA (pDNA) delivery [17,21,22]. Monoolein (MO) was originally introduced as an auxiliary lipid to aid non-viral DNA transfection in liposomal systems of dioctadecyldimethylammonium bromide (DODAB) [23]. MO is composed of an unsaturated 18-carbon alkyl chain, attached to a glycerol backbone by an ester bond (Figure 1). The amphiphilic character of MO is responsible for its self-assembly in water into cubic structures. When in mixtures with other lipids or surfactants, MO not only provides the stabilization of the liposomal structures, but also leads to the formation of reverse cubic or hexagonal phases, known to enhance fusogenicity, and, consequently, the efficient transmembrane delivery of genetic material [24,25]. Furthermore, MO is biodegradable, biocompatible, and has minimal toxicity for mammalian cells.

In this work, serine-based bis-quats (Figure 1) were used, in combination with MO, to prepare DNA carriers. The surfactants possess two long alkyl chains (12 to 18 carbon atoms) and a spacer (5 carbon atoms) linked to the nitrogen atoms of the serine residues through amine bonds. All the surfactants self-assemble in water, at room temperature, into micelles [26]. To establish the suitability of the gemini:MO aggregates as non-viral DNA vectors, their cytotoxic profile was assessed using the HEK293T cell line. The shape, size, and charge of the gemini:MO aggregates and lipoplexes (at various surfactant-to-DNA charge ratios) were evaluated by light microscopy and dynamic light scattering (DLS). The DNA complexation process was followed by DLS and quantified by fluorescence spectroscopy.

## 2. Materials and Methods

### 2.1. Materials

The enantiomerically pure *L*-O-*tert*-butyl serine methyl ester was purchased from Bachem (Bubendorf, Switzerland). Thin-layer chromatography (TLC) aluminum foil plates covered with silica 60F254 (0.25 mm), silica gel 60 (70–230 mesh ASTM) for column chromatography, solvents (p.a. quality), and chemicals (for synthesis) were obtained from Merk (Lisbon, Portugal). The serine-derived surfactants shown in Figure 1, A–pentamethylene 1,5-bis{*N*-(dodecyl)-*N*-[(1*S*)-(2-hydroxy-1-methyloxycarbonyl) ethyl]-*N*-(methyl)ammonium} trifluoroacetate, B–pentamethylene 1,5-bis{*N*-[(1*S*)-(2-hydroxy-1-methyloxycarbonyl)ethyl]-*N*-(methyl)-*N* (tetradecyl)ammonium} trifluoroacetate, C–pentamethylene 1,5-bis{*N*-(hexadecyl)-*N*-[(1*S*)-(2-hydroxy-1-methyloxycarbonyl)ethyl]-*N*-(methyl)ammonium} trifluoroacetate, and D–pentamethylene 1,5-bis{*N*-[(1*S*)-(2-hydroxy-1-methyloxycarbonyl)ethyl]-*N*-(methyl)-*N*-(octadecyl)ammonium} trifluoroacetate, were synthesized as described in the literature [26]. Monoolein (1-monooleoyl-rac-glycerol, 99% purity), used as a helper lipid, and deoxyribonucleic acid from salmon sperm (CAS: 100403-24-5) were purchased from Merck (Lisbon, Portugal).

### 2.2. Sample Preparation

Mixtures with different gemini:MO molar ratios, namely (2:1), (1:1), (1:2), and (1:4), were prepared by thin-lipid-film hydration. Briefly, gemini surfactants and MO were dissolved in ethanol to obtain 10 mM solutions. Specified volumes of each serine derivative and MO stock solution were combined, and the solvent was evaporated under vacuum at 60 °C for 45 min using a rotary evaporator. Then, the lipid films were hydrated with ultrapure (Milli-Q) water at 40 °C for 30 min, to a final surfactant concentration of 1 mM or 5 mM.

For the preparation of DNA lipoplexes, different aliquots of 1 mM aqueous solutions of the gemini–MO mixtures were added to 170 µL of the DNA solution (5.00 × 10^−4^ M) to prepare lipoplexes with gemini/DNA charge ratios ranging from 0.5 to 12. The surfactant-to-DNA charge ratio, CR (+/−), is defined as follows:

(1) $$CR\left(+/-\right)=\frac{2\times \left[gemini\;surfactant\right]}{\left[DNA\;phosphate\;groups\right]}$$

where the brackets represent molar concentrations of the respective component, considering that for gemini each molecule holds two positive charges. Lipoplexes were incubated at room temperature under vortex mixing for 1 min, and then kept under magnetic stirring for 30 min. The samples were allowed to rest for 1 h before use. The lipoplexes were then diluted with ultrapure water to a final DNA concentration of 5.7 × 10^−5^ M. The DNA concentration, measured in mM base pairs, was assessed by UV absorbance at 260 nm. The A260/A280 ratio consistently ranged from 1.8 to 1.9, confirming the absence of protein and RNA contamination.

### 2.3. Cell Culture and Cytotoxicity Evaluation

Human embryonic kidney cells (HEK293T; ATCC, Manassas, VA, USA), wild-type, were used to evaluate the cytotoxic profile of the gemini–MO aggregates at different molar ratios. The cells were grown in Dulbecco’s minimal essential medium (DMEM), supplemented with 10% heat-inactivated fetal bovine serum (FBS), 1% penicillin–streptomycin, 1% L-glutamine, and 1% sodium pyruvate (supplemented with 2 mg/mL puromycin) in a humidified incubator (5% CO_2_, 37 °C). Cells were subcultured every 2 days using 0.05% Trypsin–EDTA solution in order to maintain subconfluency.

The cytotoxicity of the mixed aggregates was assessed using the MTT assay. HEK293T cells were seeded into 96-well culture plates at a cell density of 1.5 × 10^4^ cells per well in complete cell culture medium. A 100 µL aliquot of each surfactant solution, diluted in DMEM culture medium (with a maximum H_2_O concentration of 5%) and pre-sterilized by filtration through a 450 nm membrane filter, was added to the cells at varying final concentrations (10, 25, 50, and 100 µM) and incubated. After 24 and 48 h, the medium was replaced with 80 μL of MTT solution, and the plates were incubated for an additional 2 h at 37 °C. Following this, the MTT solution was removed, and 120 μL of ethanol/DMSO (1:1) (*V*/*V*) was added to dissolve the resulting crystals. Absorbance at 570 nm was then measured using a microplate spectrophotometer (SpectraMax Plus, Molecular Devices, San Jose, CA, USA). Cell viability was calculated based on the following measurement:

(2) $$cell\;viability\;\left(\%\right)=\frac{A_{570}\;of\;treated\;cells}{A_{570}\;of\;control\;cells}\times 100$$

where the control cells were incubated with DMEM medium. The data collected for the various systems were used to generate dose–response curves, which enabled the determination of the half inhibitory concentration (IC50). This represents the concentration at which cell viability is reduced to 50% of that observed in a control culture. The results are expressed as the mean ± standard deviation (*SD*) of 3 independent experiments (*n* = 3).

### 2.4. Light Microscopy

The visualization of aggregates in the diluted solutions of gemini–MO and the phase scan studies of solid samples were carried out using an Olympus BX51 microscope in differential interface contrast (DIC) and polarized light mode, respectively. The images were obtained with a DP71 digital video camera and processed using the cellA software Version 2.x.

### 2.5. Dynamic Light Scattering (DLS)

The mean diameter <*D*_h_> and zeta potential (*ζ*-potential) of the aggregates formed in the gemini–MO mixtures, as well as in the lipoplexes, were measured with a Malvern Zetasizer Nano ZS at 25 °C. Disposable polystyrene cuvettes for size measurements and Ω-shaped capillary zeta potential cuvettes were used. For DLS and zeta potential measurements, a minimum of 5 repeats per sample were performed. In DLS, the autocorrelation function of the scattered light was fitted with Zetasizer Software^®^ v7.12 using a cumulant analysis algorithm to obtain the mean size (*z*-average) and polydispersity index. The results are presented in scattered intensity distribution data. For the ζ-potential determination, the electrophoretic mobility, *μ*, was measured using a combination of electrophoresis and laser Doppler velocimetry techniques, and the value obtained from *μ* using the Henry equation, with a dielectric constant of 78.5, a medium viscosity of 0.89 cP, and a f (κa) function value of 1.5 (Smoluchowsky approximation). The results are expressed as the mean ± standard deviation (*SD*) of 3 independent experiments (*n* = 3).

### 2.6. DNA Complexation: Ethidium Bromide Exclusion Assay

For the EtBr exclusion assays, an EtBr aqueous solution ([EtBr] = 7.0 × 10^−6^ M) was added to the DNA solution, leading to the intercalation of the probe. The EtBr concentration was kept six times lower than that of the pDNA to guarantee a decrease in the probe fluorescence directly proportional to the amount of cationic lipid at a given nucleotide base concentration [27]. The steady-state fluorescence measurements were performed, following a 5 min agitation period with a magnetic stirrer, in a Horiba Jobin Yvon Spex Fluorolog-3 spectrofluorimeter for each CR (+/−) analyzed. The fluorescence intensities were determined at λ_exc_ = 510 nm, the wavelength that is known to be an isosbestic point for EtBr/DNA solutions. All emission spectra were integrated to calculate the ratio of the areas for the dye solutions relative to the standard, after subtracting the solvent background. Each fluorescence emission spectrum was then fitted using a combination of two lognormal functions to account for the different environmental states of the probe: intercalated in the pDNA or dispersed in H_2_O [28]. Considering that the fluorescence quantum yield of EtBr in the lipoplex remains constant for all the CRs (+/−), the percentage of complexed pDNA at any [CR(+/−)*x*] can be determined from the following expression [28,29]:

(3) $$\%\;DNA=\frac{\int I_{F}\;\left(0\right)-I_{F}\;(x)}{\int I_{F}\;(0)}\times 100$$

where *I*_F_ (0) and *I*_F_ (*x*) represent the total fluorescence intensity of EtBr for a CR (+/−) = 0 (i.e., for a neat DNA solution) and for a CR (+/−) = *x* (i.e., after addition of a given aliquot of cationic aggregates to the DNA solution), respectively.

## 3. Results and Discussion

In this work, the DNA complexation ability of mixtures of serine-based gemini surfactants and monoolein was investigated. We intended to study the effect of alkyl chain length (12 to 18 carbon atoms) on the complexation and lipoplex stabilization of the mixed aggregates. The serine surfactants contain the long lipophilic chains and a five-methylene spacer, both linked to the nitrogen atoms of the serine residues by amine linkages.

### 3.1. Interactions between Gemini:MO Aggregates and DNA

**Figure 2:**
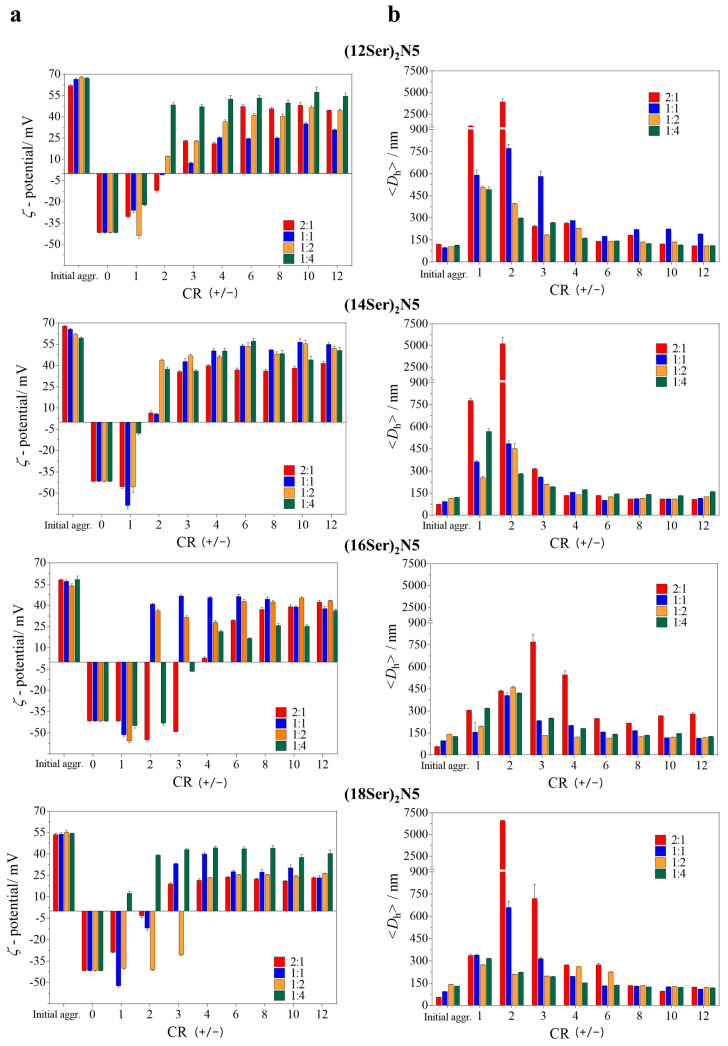
Mean *ζ*-potential ± (*SD*) (**a**) and mean diameter ± (*SD*) (**b**) (*n* = 3) of lipoplexes of gemini–MO/DNA as a function of gemini/DNA charge ratio, CR (+/−). The total gemini+MO concentration is 1 mM and the gemini:MO molar ratio in the lipoplexes varies between (2:1) and (1:4).

The gemini:MO formulations developed and their interaction with DNA was investigated. In this work, we tested mixtures with different ratios of gemini:MO (2:1, 1:1, 1:2, and 1:4) in order to study the best mixture in terms of DNA complexation and cytotoxic profile. The chosen mixing ratios are broad enough and will allow the formation of systems enriched with serine or MO, while keeping the systems more versatile. The study of lipoplex formation was monitored by measuring both the zeta (*ζ*) potential and the size of the aggregates by DLS (Figure 2). Lipoplexes were formed by adding increasing aliquots of extruded gemini:MO dispersions to a salmon sperm DNA solution to obtain different CR (+/−)values. Figure 2a,b show, respectively, the *ζ*-potential and the average diameter of the (gemini:MO)/DNA lipoplexes as a function of CR (+/−).

The *ζ*-potential measurements provide an indication of the lipoplex net surface charge that can be used to evaluate the extent of interaction of the cationic aggregate with DNA. As apparent in Figure 2a, an increasing content of cationic aggregates in the lipoplex, i.e., an increase in CR (+/−), causes an increase in *ζ*-potential relative to that of neat DNA (−41.6 ± 0.6 mV), as expected. The results show that *ζ*-potential values are both dependent on the surfactant system as well as on the MO content in the mixture.

Analyzing the lipoplexes containing (12Ser)_2_N5 and (14Ser)_2_N5, they evolve from negatively to positively charged at a CR (+/−) between 2 and 3, meaning that the charge neutrality point lies in between, and this is irrespective of the gemini:MO molar ratio. Moreover, in these systems, the *ζ*-potential attains maximal values of the order of (43 ± 9 mV), for the (12Ser)_2_N5:MO mixtures, and (48 ± 7 mV) for the (14Ser)_2_N5:MO mixtures. In contrast, both (16Ser)_2_N5 and (18Ser)_2_N5 show a more complex behavior. First, the CR (+/−) point of charge reversal varies widely with gemini:MO molar ratio within each gemini system, without a clear trend; yet, one observes that from a CR (+/−) = 4 onwards, all systems (irrespective of having C16 or C18 chains, and of MO content) are positively charged and from then onwards, the lipoplex *ζ*-potential remains, in most of the cases, constant. Additionally, the maximal *ζ*-potential attained is of (39 ± 7 mV), for the (16Ser)_2_N5:MO mixtures, and (29 ± 9 mV) for the (18Ser)_2_N5:MO mixtures, which means that for longer chain gemini the lipoplexes attain a lower maximal surface charge than for the shorter chain ones.

Concerning the variation in the lipoplex average hydrodynamic diameter with CR (+/−), Figure 2b, all systems show a kind of “bell-shaped” curve with a tail to the right of the peak (similar to a lognormal curve), as expected. The maximum <*D*_h_> in all four gemini:MO systems is of the order of 0.5–5 µm, occurring for a CR (+/−) = 1–2 for the C12 and C14 gemini systems, and a CR (+/−) = 2–3 for the C16 and C18 ones. These big µm-sized aggregates form at the effective charge neutrality of the system, which may be slightly offset from the nominal CR (+/−), and often they are unstable and precipitate out of solution with time. For a CR(+/−) ≥ 4, basically all lipoplexes (irrespective of gemini and MO content) have a mean hydrodynamic diameter in the range 150–300 nm, and low PDI values around 0.25 ± 0.05. Noteworthily, in the case of (18Ser)_2_N5:MO lipoplexes, already for a CR (+/−) ≥ 2 when the MO content is high, (1:2) and (1:4), the lipoplexes remain below 200 nm with low polydispersity (PDI = 0.20 ± 0.01).

**Figure 3:**
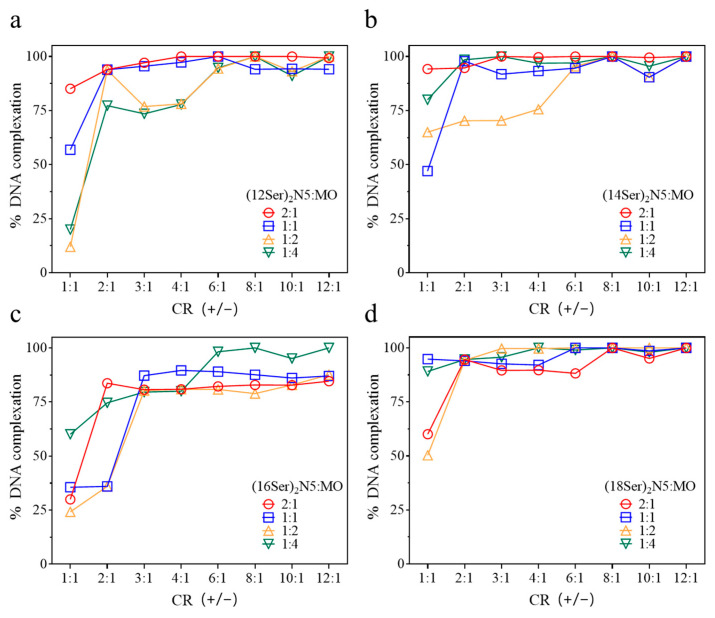
Percentage of complexed DNA for gemini:MO lipoplexes, at different gemini/DNA charge ratios, CR (+/−). (**a**) (12Ser)_2_N5:MO/DNA systems, (**b**) (14Ser)_2_N5:MO/DNA systems, (**c**) (16Ser)_2_N5:MO/DNA systems, and (**d**) (18Ser)_2_N5:MO/DNA systems.

The DNA complexation efficiency of the gemini aggregates was assessed using fluorescence spectroscopy with ethidium bromide (EtBr) as the fluorescence dye. EtBr, a well-established DNA probe, intercalates into the DNA double helix. When cationic aggregates bind electrostatically to DNA/EtBr, EtBr is displaced from the DNA into the solution, leading to a quenching of its fluorescence. This decrease in fluorescence intensity can be expressed in terms of percentage compared to the initial maximum EtBr fluorescence in DNA, giving an indication of the amount of condensed DNA at different CRs (+/−). As can be seen in Figure 3, the pattern of complexation is complex among the systems, but some main trends can be observed.

It is clear that the (18Ser)_2_N5 system is overall the most efficient one, showing percentages of DNA complexation up to 100% for a CR (+/−) ≥ 2 and for all gemini:MO molar ratios. On the other hand, the (16Ser)_2_N5 mixtures show, in general, the lowest capacity to complex DNA, namely for gemini:MO ratios of (2:1), (1:1), and (1:2). Only the mixture with the highest content of MO (1:4) presents percentages of complexed DNA up to 90%; however, this happens only for a charge ratio ≥ 6:1. For the shorter chain gemini (C12 and C14), the highest complexation efficiencies (up to 100%) are attained for mixtures with the lowest content of helper lipid, (2:1) and (1:1), and charge ratios ≥ 2. In particular, for the (12Ser)_2_N5 system, mixtures with higher content of MO (1:2 and 1:4) were the least effective for DNA complexation, showing percentages above 87% only for charge ratios above 6:1.

### 3.2. Characterization of the Gemini:MO Aggregation Behavior

**Figure 4:**
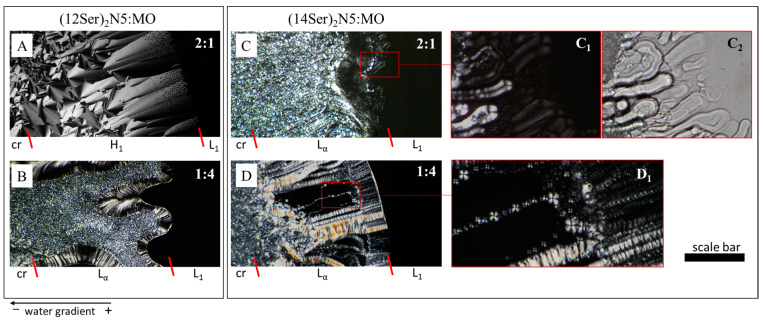
Representative phase penetration scans for (12Ser)_2_N5:MO (**A**,**B**) and (14Ser)_2_N5:MO (**C**,**D**) at molar ratios 2:1 and 1:4, respectively. Water is diffusing from right to left into the surfactant crystalline film. Legend: L_1_, isotropic solution phase; H_1_, hexagonal phase; L_α_, lamellar phase; cr, hydrated crystals. Scale bars: (**A**–**D**) 150 µm; (**C1**,**C2**) 80 µm; and (**D1**) 50 µm.

To obtain insight into the types of aggregates and the lyotropic phase behavior of the gemini:MO mixtures, phase penetration scans were performed on a polarized light microscope. Two types of behavior were observed. The mixtures based on (12Ser)_2_N5 show a phase behavior dominated by a hexagonal phase (Figure 4A), except for the (1:4) system. For the latter molar ratio, a dilute isotropic solution phase, L_1_, is followed by a lamellar liquid crystalline phase, L_α_, characterized by oily streaks. In contrast, the mixtures based on gemini surfactants with longer chains—(14Ser)_2_N5, (16Ser)_2_N5 and (18Ser)_2_N5—exhibit similar phase behavior for all MO contents, where the isotropic L_1_ phase is followed by a lamellar phase, L_α_, characterized by oily streaks (Figure 4C,D), myelin figures (Figure 4C,C1,C2), and focal conics (Figure 4D,D1).

**Figure 5:**
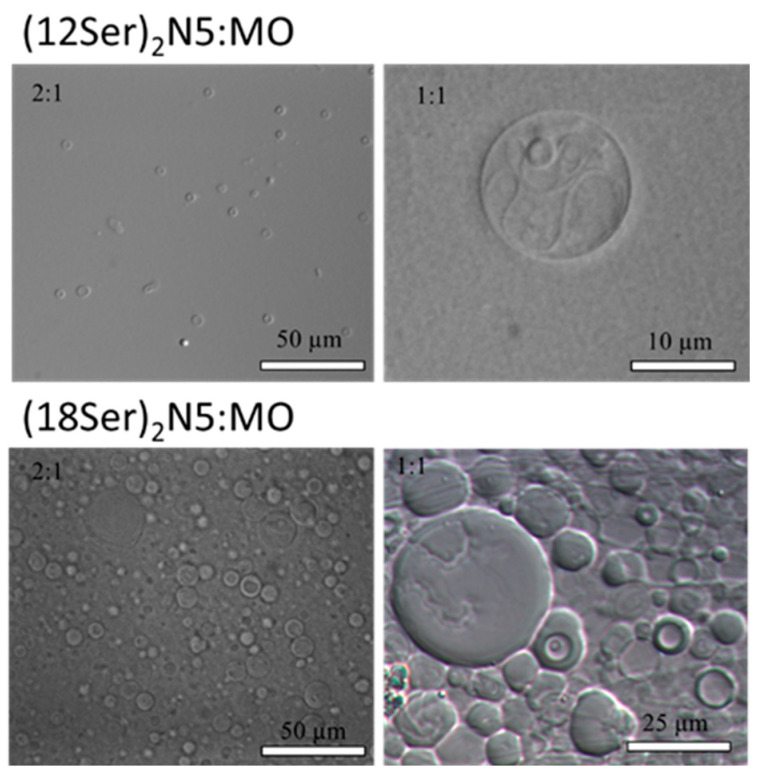
Representative imaging of (12Ser)_2_N5:MO and (18Ser)_2_N5:MO vesicles by light microscopy, for different gemini:MO molar ratios, for 10 mM dispersions.

Another significant observation is that the inclusion of MO leads, in most of the mixtures, and at low concentrations, to the formation of vesicular dispersions (Figure 5). The vesicular structures are spherical and very polydisperse, with sizes ranging from 0.5 to 10 µm and occasionally with the presence of a few multivesicular aggregates and very big structures (>20 µm), as assessed by light microscopy.

### 3.3. Discussion of the Main Interaction/Aggregation Trends

DNA complexation is primarily driven by electrostatic interactions between the DNA phosphate groups and the cationic surfactant headgroups. This electrostatic attraction neutralizes the charge on the DNA, facilitating its complexation and subsequent compaction. The thermodynamic driving force behind the DNA/surfactant lipoplex involves two main factors: direct Coulombic attraction between the charges of the two cosolutes, leading to a favorable enthalpic effect (∆*H* < 0), and the release of counterions from the surfactant aggregate and DNA surface, resulting in a favorable entropic effect (∆*S* > 0). Electrostatic interactions with DNA are inherently favored for the neat gemini aggregates, which possess two cationic charges per headgroup. The addition of monoolein to these cationic aggregates may further enhance such interactions, as presumably MO weakens the ion binding to the cationic surface, increasing the availability of positively charged sites for DNA binding. Another important factor to consider in surfactant/DNA complexation is the role played by hydrophobic interactions. The latter occur between the hydrophobic tails of the surfactants and the hydrophobic regions of DNA (such as the bases), aiding in the complexation, compaction, and stabilization of the DNA–surfactant aggregates. Herein, the role played by such interactions is evident in the fact that the longest chain surfactant, (18Ser)_2_N5:MO, the most hydrophobic one, shows very high complexation efficiencies and forms small lipoplexes even at charge ratios as low as 2, and irrespective of monoolein content.

All the serine-based gemini compounds used in this work are soluble surfactants, forming micelles at concentrations in the order of 1–320 µM [26]. These gemini compounds have a *n*-5-*n* molecular structure, with *n* = 12, 14, 16, and 18. The critical packing parameter of a surfactant, *P*_s_, is defined as the ratio (*V*_hc_/*a*_hg_ lhc), where *V*_hc_ and *l*_hc_ are the volume and length of the surfactant hydrocarbon chain and *a*_hg_ is the optimal headgroup area at the aggregate interface. The relatively short and hydrophobic 5-methylene spacer tends to remain at the surface of the formed aggregate, causing the surfactant to adopt a cone-shaped geometry, i.e., a critical packing parameter, *P*_s_, of the order of 0.33 [30], and hence the formation of small (possibly spheroidal) micelles [31]. MO has a *P*_s_ > 1 and tends to form reverse structures (namely cubic phases). The inclusion of MO leads to the formation of a lamellar liquid crystal phase in most of the mixtures, and, at low concentrations, to vesicular dispersions, structures for which *P*_s_ has an intermediate value of 0.5–1. Since the lipoplexes result from the interaction between these bilayer structures with DNA, this suggests (though obviously does not prove) that lipoplexes may also have a lamellar structure. In lamellar lipoplexes, surfactant/lipid molecules form bilayers with DNA intercalated between them, in an arrangement that is akin to the structure of biological membranes. In the case of the (12Ser)_2_N5:MO system, at molar ratios (2:1), (1:1), and (1:2), the incorporation of MO results in the formation of a hexagonal liquid crystal phase. We may thus also speculate that in these cases the lipoplexes may consistently adopt hexagonal structures, where DNA would compact between the cylindrical micelles (for a normal hexagonal phase) or inside them (for a reverse hexagonal phase). To fully elucidate the structure of the lipoplexes, other techniques must be used, namely small-angle X-ray scattering (SAXS) and cryo-transmission electron microscopy.

### 3.4. Cytotoxicity of the Gemini–MO Systems

**Figure 6:**
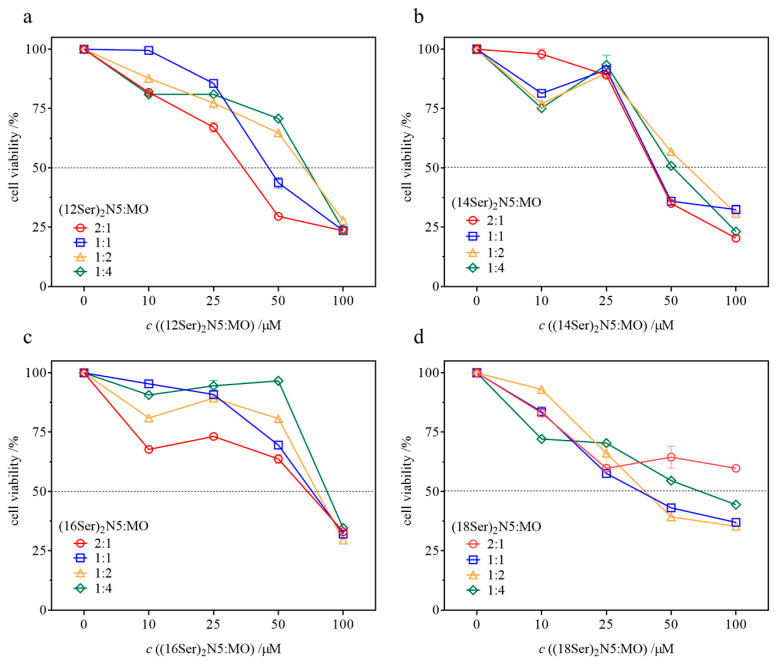
Dependence of cell viability ± standard deviation (*SD*) (*n* = 3) with increasing gemini:MO concentration after 48 h of exposure, for different gemini:MO molar ratios, from (2:1), (1:1), (1:2) to (1:4). (**a**) (12Ser)_2_N5:MO systems, (**b**) (14Ser)_2_N5:MO systems, (**c**) (16Ser)_2_N5:MO systems, and (**d**) (18Ser)_2_N5:MO systems.

The evaluation of the cytotoxic profile of novel DNA-compacting aggregates is a fundamental step towards an initial screening of the most promising systems as prospective gene delivery vectors. Cytotoxicity was assessed in human embryonic kidney 293 cells, in an amphiphile (gemini + MO) concentration range of 10 to 100 µM for 48 h, yielding the results shown in Figure 6. The IC_50_ values (minimum concentration to promote a 50% decrease in cell viability) of all systems are summarized in Table 1. From the data presented in Figure 6, a few trends can be observed. First, there is a decrease in cell viability with increasing gemini:MO total concentration, as expected, down to around 25%, from which IC_50_ values can be obtained. Second, as the content of MO in the mixtures increases, there seems to be a general tendency for the toxicity of the formulations to decrease (rise in IC_50_), even though this trend is not always observed, given the uncertainties in IC_50_. Third, formulations containing the shorter chain gemini surfactants, C12 and C14, seem to be in general more cytotoxic than those with longer chains (C16 and C18), though again this trend is not always observed. Finally, and most significantly, for concentrations below ~35 µM, most of the systems show cell viabilities higher than 70% and therefore, according to the international ISO guidelines, induce low or no cytotoxicity [32]. It should be highlighted that for transfection experiments the necessary surfactant:lipid mixture concentrations are typically below 20 μM, and therefore these aggregates are not cytotoxic at the concentration range needed.

## 4. Conclusions

In this study, we investigated cationic serine-based gemini surfactants for their cytotoxicity and efficacy in complexing and compacting DNA, in mixtures with monoolein (MO) as a helper lipid. Significantly, the cytotoxicity assays unveiled that all the gemini–MO systems show IC_50_ values above 35 µM, underscoring good viability, and the physicochemical studies showed that these systems can efficaciously complex DNA, attaining percentages of around 90–100%. Moreover, the size of the resulting lipoplexes decreases as the charge ratio, CR (+/−), increases, until it reaches a plateau. A non-monotonic variation in the lipoplexes’ mean size is observed. In general, a maximum is attained for a CR (+/−) = 2 or near it. Typically, for a CR (+/−) ≥ 4 a plateau is reached, with the lipoplexes having a similar size as the initial aggregates, of the order of 150–300 nm. Furthermore, for all gemini:MO systems, the formed lipoplexes (for a CR (+/−) ≥ 4) are positively charged, reaching values in the range of + 20 to + 55 mV, and this might aid in their colloidal stability and capacity to penetrate cell membranes. From phase penetrations scans, it is shown that the gemini:MO systems with longer alkyl chains (14, 16, and 18 carbon atoms) form a dilute isotropic L_1_ phase, followed by a lamellar liquid crystalline phase (L_α_). Vesicles are also observed for these systems at low concentrations (10 mM), suggesting that the lipoplexes may also consist of lamellar structures. In contrast, the (12Ser)_2_N5:MO systems exhibit a phase behavior dominated by a hexagonal phase (with the exception of the (1:4) system that forms a L_α_), which could entail lipoplexes also based on a hexagonal structure. To conclude, this work paves the way for the design and implementation of gene transfection studies based on gemini:MO aggregates to more comprehensively assess their potential as gene delivery vectors.

## Figures and Tables

**Table 1:** IC_50_ ± standard deviation (*SD*) (*n* = 3) values determined by MTT assay in HEK293T cell line upon exposure to aqueous mixtures of gemini:MO, at varying molar ratios, for a gemini:MO concentration ranging from 10 to 100 µM.

System	Gemini:MO Molar Ratio	IC_50_/µM
(12Ser)_2_N5:MO	2:1	35 ± 5
	1:1	50 ± 5
	1:2	62 ± 9
	1:4	64 ± 5
(14Ser)_2_N5:MO	2:1	45 ± 6
	1:1	51 ± 8
	1:2	62 ± 7
	1:4	55 ± 8
(16Ser)_2_N5:MO	2:1	64 ± 3
	1:1	72 ± 3
	1:2	76 ± 7
	1:4	90 ± 7
(18Ser)_2_N5:MO	2:1	>100
	1:1	43 ± 7
	1:2	46 ± 8
	1:4	74 ± 8

## Data Availability

The raw data supporting the conclusions of this article will be made available by the authors on request.
